# A Novel Extra Tree Ensemble Optimized DL Framework (ETEODL) for Early Detection of Diabetes

**DOI:** 10.3389/fpubh.2021.797877

**Published:** 2022-02-15

**Authors:** Monika Arya, Hanumat Sastry G, Anand Motwani, Sunil Kumar, Atef Zaguia

**Affiliations:** ^1^Department of Computer Science and Engineering, Bhilai Institute of Technology, Durg, India; ^2^School of Computer Science, University of Petroleum and Energy Studies, Dehradun, India; ^3^School of Computing Science and Engineering, VIT Bhopal University, Sehore, India; ^4^Department of Computer Science, College of Computers and Information Technology, Taif University, Taif, Saudi Arabia

**Keywords:** data stream classification, deep learning, diabetes detection, ensemble technique, extra tree ensemble, machine learning, overfitting, feature selection

## Abstract

Diabetes has been recognized as a global medical problem for more than half a century. Patients with diabetes can benefit from the Internet of Things (IoT) devices such as continuous glucose monitoring (CGM), intelligent pens, and similar devices. Smart devices generate continuous data streams that must be processed in real-time to benefit the users. The amount of medical data collected is vast and heterogeneous since it is gathered from various sources. An accurate diagnosis can be achieved through a variety of scientific and medical techniques. It is necessary to process this streaming data faster to obtain relevant and significant knowledge. Recently, the research has concentrated on improving the prediction model's performance by using ensemble-based and Deep Learning (DL) approaches. However, the performance of the DL model can degrade due to overfitting. This paper proposes the Extra-Tree Ensemble feature selection technique to reduce the input feature space with DL (ETEODL), a predictive framework to predict the likelihood of diabetes. In the proposed work, dropout layers follow the hidden layers of the DL model to prevent overfitting. This research utilized a dataset from the UCI Machine learning (ML) repository for an Early-stage prediction of diabetes. The proposed scheme results have been compared with state-of-the-art ML algorithms, and the comparison validates the effectiveness of the predictive framework. This proposed work, which outperforms the other selected classifiers, achieves a 97.38 per cent accuracy rate. F1-Score, precision, and recall percent are 96, 97.7, and 97.7, respectively. The comparison unveils the superiority of the suggested approach. Thus, the proposed method effectively improves the performance against the earlier ML techniques and recent DL approaches and avoids overfitting.

## Introduction

In a survey made by IDF and WHO, nearly half a billion of the population worldwide have diabetes and posing about $13,700 financial burden per year. Moreover, the statistics increasing in years to come. Diabetes is a persistent disease caused when the blood sugar level crosses certain levels and can have adverse consequences on other organs of the human body and severely affect the entire body. If it is not diagnosed at the right time and remains untreated, it can increase the risk of other disorders. In addition, diabetes may lead to other problems like kidney failure, weak eyesight and heart problem.

Furthermore, diabetic persons are at higher risk of infection and death from COVID-19 (1). Hence, concerning its severe complications, prior diagnosis of the disease is very significant to take timely steps to avoid other health risks and complications. Patients with diabetes can benefit from the Internet of Things devices such as CGM, intelligent pens, and other similar devices, according to the American Diabetes Association (ADA). These devices help in collecting medical data in real-time. Data collected from the smart devices needs to be processed immediately for dissemination of information to practitioners to provide prompt medical attention. Health care data comes from various sources, including medical history and records of patients in hospitals, medical diagnosis reports, medical examination reports by doctors, real-time data from multiple IoT devices and health-related Apps, and data streams from social networking sites. Dealing with such heterogeneous healthcare data has become increasingly difficult in recent years, owing to the large volume of data, security issues, incompetence in wireless network application development, and the rapidity with which it is being generated. As a result, to improve the healthcare industry's efficiency, accuracy, and workflow, data analytics tools are required to manage such complex data (2). Therefore, it is necessary to process this streaming data faster to obtain relevant and significant knowledge using an adaptive model. In the recent past, many algorithms have been employed in handling the classification of real-time data streams. Gaining insights and knowledge from such streaming data is a crucial challenge. Machine Learning (ML) is an analytical method for mechanized learning. It learns from knowledge and skill gained during the training process and applies the experience to improve the performance to make more accurate predictions. ML plays a significant role in discovering hidden and new patterns in the medical data stream. These patterns provide fascinating insights into the knowledge gained.

Further, researchers and medical practitioners use it for assistance in various ailment diagnoses and treatments. Latest advances in this field can also be applied for discovering unknown and latent patterns for detecting diabetes in prior stages. The advancement in ML techniques resolves this censorious problem of an early diabetes diagnosis.

Only health professionals are permitted to process health data because it is sensitive and not readily available. In addition, it is subject to strict usage rules due to the obligation of medical secrecy. As a result, only health professionals are permitted to process it. ML algorithms frequently underperform in prediction accuracy when there is insufficient data to train the model. The data obtained from heterogeneous sources can be either structured or unstructured, depending on the source. Conventional ML techniques cannot process unstructured data; on the other hand, DL can analyze images, videos, and unstructured data in ways that traditional ML techniques cannot. Deep understanding, as opposed to ML, typically necessitates less ongoing human intervention. DL models for prediction and classification are becoming increasingly popular as a means of avoiding these pitfalls.

DL is a subset of Artificial Intelligence with similar architecture as a neural network but has extra hidden layers. The extra layers, therefore, make DL more powerful in data processing than shallow architecture. DL methods showed more accuracy in results than traditional rule-based methods in various domains, including eHealth systems (3–5). A study proposed a hypothesis that further improving the accuracy of DNN feature selection techniques can be used (6, 7). Feature selection is the process of acquiring relevant information and discarding irrelevant ones (8). The feature selection methods can be either supervised or unsupervised, and the supervised method can be divided into the wrapper, filter or intrinsic methods.

Furthermore, a single feature selection method may produce a local optimal or sub-optimal feature subset for which a learning method's performance is compromised. Therefore, multiple feature subsets are combined in the ensemble-based feature selection method to select an optimal subset of features using a combination of feature ranking that improves classification accuracy. The normalized total reduction is the mathematical criteria used in the split decision during the forest construction when the extra tree ensemble method performs feature selection. In addition, in the proposed work Gini Index is computed for each feature known as the Gini Importance of the feature. Then, each feature is ordered in descending order based on its Gini Importance, and the user selects the top k features based on the preferences to perform feature selection. This process selects the optimal feature subset from the high dimensional feature space.

However, the DNN has a disadvantage because it overfits small data sets (9, 10). Overfitting occurs when the accuracy with the training dataset is greater than the accuracy with the testing data set, and the model is not generalized. Dropouts can avoid this problem where a certain number of neurons at a layer are deactivated from firing during training. This deactivation of neurons prevents overfitting, and the network's performance on test data improves (11).

The novelty of the proposed work is as follows:

- The Extra tree ensemble feature selection technique reduces the feature space by selecting the optimal feature subset. Thus, improving the prediction accuracy and reducing model complexity.
- When presented to the DL network, the optimal feature subset further enhances its performance and prevents overfitting.
- Previous works are either complex or prone to overfitting. Both these issues are fixed in the proposed work.

Following are the key contributions of the Novel ETEODL framework.

- The proposed algorithm predicts diabetes at a very early stage using a framework that combines an extra-tree ensemble feature extraction technique used to extract relevant features and a deep neural network to improve prediction accuracy.
- Valuable metrics: prediction accuracy, Precision, Recall, F1-score, and computation time are evaluated for performance comparison.
- The proposed algorithm is compared with state-of-the-art techniques.
- The proposed algorithm shows no instances of overfitting or underfitting.

## Literature Review

Alić et al. (12) studied several diverse ML techniques to detect diabetes and concluded that the most common type of ANN used is multilayered feed-forward and the Naïve Bayesian network, which shows higher possibilities of get-ting accurate predictions. In their work, Sisodia and Sisodia (13) experimented with Decision Tree, SVM, and Naive Bayes and concluded that Naive Bayes has the highest accuracy compared to other algorithms and verified it using ROC metrics. Maniruzzaman et al. (14) used Gaussian process classification (GPC) to diagnose diabetes and concluded that the model's performance is comparatively better than other models. In their work, Kaur and Kumari (15) analyzed different models for the detection of diabetes. Wei et al. (16) explored the popular techniques to detect diabetes and data pre-processing techniques. Kamble et al. (47) proposed a DL-based Restricted Boltzmann machine approach for detecting diabetes. Swapna et al. (17) used DL-based methods to classify diabetic and HRV signals by extracting dynamic features from HRV data using a combination of LSTM and CNN. However, LSTM has high computational complexity and is prone to overfitting (18). Yahyaoui et al. (19) compared traditional classifiers with DL-based classifiers, Random Forest (RF) shows more accurate results in predicting diabetes than DL and SVM methods. DL (DL) techniques like CNN and RNN improve performance compared with classic designs (20). Naz and Ahuja (21), in their work, concluded that DL approaches perform better for early detection of diabetes as compared to Artificial Neural Network (ANN), Naive Bayes (NB), and Decision Tree (DT). The DL techniques also facilitate the latest trending techniques like Edge AI applications (22). A regularization layer called dropout can be used in fully connected layers of DNN to address the problem of overfitting (23). Rubaiyat et al. (24) used feature selection and used the selected features with traditional ML techniques like Random Forest, Logistic Regression, and MLP neural network classifier. Iwendi et al. (25) proposed a system to improve intrusion detection using a combination of correlation-based feature selection techniques and machine learning ensemble models. Reddy et al. (26) proposed ensemble-based ML algorithms, compared the proposed performance against the individual ML algorithms and concluded their superiority over the unique ML algorithms. Bashir et al. (27) used ensemble techniques for diabetes detection and inferred that the Bagging ensemble outperforms other ensemble techniques.

Similarly, Tama and Rhee (28) concluded that a tree-based classifier is better than other approaches. Recent research has been concentrated on enhancing the performance of ensemble-based methods for the prediction of disease. In addition, the neural network-based models can further reduce the cost (29). Deepa et al. (30) proposed RASGD to improve the regularization of the classification model. It is done by employing weight decay methods, such as the least absolute shrinkage and selection operator. In addition, ridge regression methods are used to achieve better regularization. Gadekallu et al. (31) were motivated by the fact that previous work had neglected the aspects of data pre-processing and dimensionality reduction, which had resulted in skewed results. Consequently, in their work, the raw dataset is normalized using the Standardscalar technique and Principal Component Analysis (PCA) is used to extract the most significant features from the dataset. In addition, the Firefly algorithm is used to reduce the dimensionality of the data. Finally, this condensed dataset is fed into a Deep Neural Network Model, used to classify the data. Gadekallu et al. (32), in their work, use a principal component analysis-based deep neural network model with the Gray Wolf Optimization (GWO) algorithm. The application of GWO allows for the selection of the most optimal parameters for training the DNN model.

## Research Motivation

In this era of big data, various types of biomedical data for early detection of diabetes have been emerging from electronically generated health records, medical images, IoT sensor data, and simple text data. This streaming data is intricate, diverse, appallingly annotated, and commonly not structured. Early detection of the disease is significant to take timely steps to avoid other health risks and complications. However, the earlier studies depict that the ML algorithms need structured data for classification, have less prediction accuracy, prone to overfitting, and require more computational time to predict the disease. While DL models are more promising, as DL networks are flexible, making them suitable for structured and unstructured data (33), they can process the data than the shallow architecture.

Further, their accuracy can be improved by appropriate feature selection techniques (34, 35). Thus, DL approaches could employ big biomedical data to improve human health (36). For example, most of the recent research (20) concluded that DL performs better for early detection of diabetes as compared to Artificial Neural Network (ANN), Naive Bayes (NB), and Decision Tree (DT).

In literature, various techniques like linear regression feature selection, logistic regression feature selection, Correlation-value based feature selection, Chi- square-based feature selection, F-score based feature selection, decision tree feature selection and random forest feature selection are used for selecting relevant features.

Table 1 below summarizes the methodology used and the limitations of some of the pertinent recent works.

**Table 1:** Methodology and limitations of recent relevant work.

**S.No**	**Author**	**Methodology used**	**Limitations**
1	Cho et al. (37)	A model which combines Linear SVM classifiers and wrapper or embedded feature selection methods	Wrapper methods for feature selection have high computational costs and are generally prone to overfitting. They are also dependent on the classifiers used.
2	Le et al. (38)	A novel model utilizing Gray Wolf Optimization (GWO) and an Adaptive Particle Swam Optimization (APSO) to optimize the Multilayer Perceptron (MLP) to reduce the number of required input attributes.	In MLP, computations are complex and time-consuming.
3	Lukmanto et al. (39)	A classification framework to identify and classify diabetes datasets using F-Score Feature Selection and Fuzzy SVM.	A disadvantage of the F-score is that it does not reveal mutual information among features. Instead, it only captures the linear relationships between features and labels.
4	Putri et al. (40)	Learning Vector Quantization (LVQ) to classify the diabetes dataset with Chi-Square for feature selection.	Chi-Square for feature selection does not take into consideration the feature interactions. It is best suited only for categorical variables
5	Sneha and Gangil (41)	Classification by selecting the optimal features based on the correlation values.	Correlation values for feature selection uncover only relationships and do not determine what variables have the most influence. Thus, it can be a time-consuming process.

Chen et al. (42) concluded that feature extraction might improve the performance of deep neural networks. In their work, Motwani et al. (43) suggested a framework based on a deep neural network for intelligent patient monitoring. Motwani et al. (44) used DL with cost optimization for remote patient monitoring and recommendation. Authors in (45) compared various feature selection techniques and concluded that the random forest algorithm performs better than the other algorithms. Trapping this advantage of the tree-based technique for selecting relevant features based on feature importance, the proposed approach uses the Extra tree ensemble feature selection technique to retrieve the optimal feature set. Furthermore, a single feature selection method may produce a local optimal or sub-optimal feature subset for which a learning method's performance is compromised. Therefore, multiple feature subsets are combined in the ensemble-based feature selection method to select an optimal subset of features using a combination of feature ranking that improves classification accuracy.

Thrust by these facts, this paper proposed an Extra-Tree Ensemble optimized DL framework (ETEODL) to predict the likelihood of diabetes. This approach is a combination DL approach for prediction and an Extra Tree ensemble technique for selecting the best features based on feature importance. The DL approach extracts lower-level information and feeds them to the next higher layer. The dropout technique is used with hidden layers of DNN to prevent the overfitting of the model.

The proposed model performs better in the following aspects:

1. Prediction accuracy and computational time
2. Prevent overfitting.

The framework is compared with traditional ML techniques and recent works for various parameters like Accuracy, F1-Score, Precision, Recall, and Computation time and thus concluded that the efficiency of the proposed framework is better than the compared works.

The paper has the following sections. First, in section Methodology and Algorithm of the proposed framework are discussed. Then, in section 5, Experimental Setup, including Dataset description, Experiment Environment setup, Experiments, and results, has been discussed. In the last, the proposed work is concluded, and directions for future research are suggested.

## Methodology and Algorithm

The DL framework used in this paper for the early prediction of diabetes is called ETEODL. The framework is divided into two segments. The first segment performs the data acquisition, pre-processing, and feature extraction, while the second segment performs the prediction using a DL model. First, data is acquired from the UCI repository. The acquired data is pre-processed to make it ready for further processing. Next, the data set is split for training and testing purposes. It is followed by feature extraction, where relevant features are extracted to reduce the data set's feature space and prevent model overfitting. The extra tree feature extraction technique is used in the proposed work. The output of the first segment is fed in a DL model for classification and prediction. The functions of the two segments are explained in detail in the following section.

### Segment-1

#### Data Acquisition and Data Pre-processing

Data is acquired and pre-processed in the first phase of the framework to clean, transform, and reduce the dimensions. Then, the missing values are discarded. Finally, the normalization process does data transformation. The dataset is divided in a ratio of 80:20 for training and testing purposes for ensuring the learning process only from the training data. After training the model, the performance is tested using testing data.

#### Data Feature Selection Using Extra Tree Ensemble

After data pre-processing, feature selection is made using Extra Tree Ensemble technique. In this step, the subset of the most relevant features is selected. The choice of the most pertinent features influences the model performance greatly. In the proposed framework, feature importance property is utilized for feature selection. In this method, each feature is given a score. The score ranges from zero and one. The leading score indicates more relevancy of the feature toward the targeted output. These relevant features are thus chosen for model building with improved predictive accuracy and controlled over-fitting. A subset of randomly selected n features is supplied to each test node of the tree. Further, the best feature is chosen by the decision tree from this subset to split the data based on Gini Index. The output features of segment one are used as input in segment 2.

### Segment-2

#### DL Model With Dropouts

The DL model consists of three types of layers:

The Input layer is where the selected features are passed. No computation occurs in this layer. The Hidden layers are present between the input and output layers. For choosing the number of Hidden Layers following basic rules are followed;

1. If the data is linearly separable, then no hidden layers are required.
2. Using neural networks with one to two hidden layers would be appropriate if the data is less complex and has fewer dimensions or features.
3. If the data has many dimensions or features, it is possible to use three to five hidden layers to achieve the best possible result.

The input data is not linearly separable and is complex as obtained from various heterogeneous sources; the proposed model consists of three hidden layers. The hidden layers one and two utilize the Rectified Linear Unit (ReLU) as activation.

Mathematically, it is defined as


(1) $$\begin{array}{r} y=max\left(0,x\right) \end{array}$$

And at hidden layer three uses the sigmoid as an activation function. The sigmoid is mathematically represented as:


(2) $$\begin{array}{r} f\left(x\right)=1/\left(1+e^{\wedge }\left(-x\right)\right) \end{array}$$

These hidden layers perform the computation and pass the information to the output layer in the end.

The output layer is responsible for producing the out variable and giving the result.

The cost or loss function is binary cross-entropy/log loss represented using E(W).


(3) $$[E\left(W\right)]=-\;\frac{1}{m}\sum_{i=1}^{m}y_{i}\log\left(\gamma \right)+(1-y_{i})\log\left(1-\gamma \right)$$

**Figure 1:**
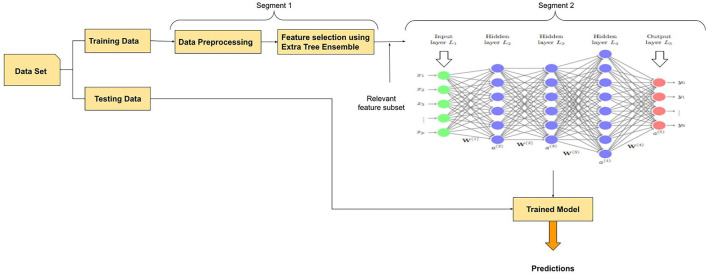
Flowchart for the proposed framework.

A dropout layer is added to the model after each hidden layer to prevent overfitting. During the training phase, the dropout layer deactivates a random set of fractions “i” neurons. The value of *p* is set to 0.8, where *p* is the probability of retention used in the input layers and is set to 0.5 in the hidden layers. The value of c is set to 4 in all the layers, where c is the Max-norm constraint. The step-wise methodology of the framework is shown in Figure 1.

The algorithm of the proposed framework is given in below.

**Algorithm 1:** Extra Tree Ensemble Optimized DL Algorithm

*Input: X:* Training Data set ỹ	
	*Y:* Class Label of *X*
	*x:* Unknown sample
*Output:* Label k for unseen sample x	
1: Call Algorithm-2 for ETE on Dataset X;	
2: Ensemble *x*_*n*_ = *h*(ŷ_1_, ŷ_2_, ……ŷ_*n*_)	
3: Transform Input Features: *x*_*n*_ *to Tensor Tx*_*n*_	
4: for each hidden layer, l do	
	a. *r*_*j*_ ( *l* )~ *Bernaulli* (*p*)
	b. ỹ( *l* ) = *r* ( *l* )^*^y (*l*)
	c. $z_{i}\;\left(\;l\;+\;1\;\right)\;=\;w_{i}\;\left(\;l\;+\;1\;\right){\;}^{*}ỹ\;\left(\;l\;\right)\;+\;b_{i}\;\left(\;l\;+\;1\right)$
	d. *y*_*i*_ ( *l* + 1 ) = *f* (*z*_*i*_ ( *l* + 1 ))
5: Calculate the probability score for predicting the class of transaction: $$\begin{array}{l} ŷ=\sigma \left(\;Wo\;\left[Td_{m}\;\right]+\;b_{f}\;\right) \end{array}$$	
6: Calculate objective function such as Error Function: E(W) is calculated as $[E\left(W\right)]=-\;\frac{1}{m}\sum_{i=1}^{m}y_{i}\log\left(\gamma \right)+(1-y_{i})\log\left(1-\gamma \right)$	
7: Predict diabetes for the given feature set.	

**Algorithm 2:** ETE

*Input: F*
*Output: A Split [f*<*fc] or none*
1: if (|*F*| < *n*_min_) then
return SS=True;
2: if all features are constant in F
return SS=True;
3: if the output is constant in F
return SS=True;
else
return SS=false;
Endif
4: if (SS) is TRUE
return none;
else
a) Select k features {*f*_1_, *f*_2_, …………..*f*_*k*_} from all *f*_*c*_ that is variable (in F);
b) Take k splits {*s*_1_, *s*_2_, …….*s*_*k*_ } where *s*_*i*_ = call Algorithm-3 Rand_Split (s, f_i_) ∀ _i_=1,2,...k;
c) return a split s^*^ such that Score (s^*^, F) = max i=1 to k Score (s^*^, F);
End if

**Algorithm 3:** Rand_Split(s,f)

*Input: s and f*
*Output: a split*
1: Draw a random cut point *f*_*k*_ uniformly in [*fs*_min_, *fs*_max_];
2: return the split [*a*<*a*_*c*_];

## Experimental Setup and Results

### Data Set

The dataset used in the research is taken from the UCI repository (34). It contains 520 instances and 16 attributes. The missing values have been pre-processed by discarding the tuples with incomplete values.

### Experiment Environment Setup

The experimental setup includes an Intel Core i5 processor with 16 GB RAM. The software configuration includes Keras, Google Tensorflow, and other required libraries such as Scikit-Learn, Numpy, and Pandas installed over Python.

### Results and Discussion

The proposed ETEODL model was implemented over Python. The parameters to evaluate the model. The model efficiency was evaluated based on essential metrics like prediction accuracy, precision, recall, f1-measure, ROC, and RMS Error (14). The proposed model is compared with conventional techniques (13) like Naïve Bayes, Decision Tree, Random-forest, Hoeffding tree, and ensemble classifier-like stacking and also with the recent three related works (17, 18, 46). The comparisons are with conventional work is summarized in Table 2, and comparison with recent existing work is summarized in Table 3.

**Table 2:** Comparison of proposed model with conventional ML algorithms.

**Classifier**	**Accuracy%**	**Precision %**	**Recall %**	**F1-score %**	**ROC area %**	**Comp time**
Naïve bayes	87.5	88.2	87.5	87.6	94	38.65
Decision tree	80.76	85.3	80.7	81.1	83.7	45.56
Hoeffding tree	87.5	88.2	87.5	87.6	94	50.68
Random forest	95.19	95.55	95.19	95.2	91.1	54.72
Ensemble (stacking)	63.46	83.4	63.46	73.5	50	69.8
ETEODL (proposed)	97.38	97.7	97.7	96	95	28.63

**Table 3:** Comparison of the proposed model with recent works.

**Title**	**Methodology**	**Accuracy**	**Precision**	**Recall**	**F1-score**	**ROC area**	**Computation time**	**Limitation/drawback**
Diabetes detection using DL algorithms (17)	Employed long short-term memory (LSTM), convolutional neural network (CNN), and its combinations for extracting complex temporal dynamic features	95.7	0.77	0.86	0.87	0.94	58.73	LSTM have high computational complexity and is prone to overfitting
Health care system: stream ML classifier for features prediction in diabetes therapy (46)	Used combination of probabilistic and ML models	90	0.746	0.678	0.85	0.5	45.67	The Probabilistic approach suffers from the problem of selecting the suitable metrics to conduct a detection process
Diabetes detection using DL approach (47)	DL-based Restricted Boltzmann machine approach is used.	84.32	0.86	0.75	0.77	0.911	67.83	In RBM, training is more problematic as it is difficult to calculate the energy gradient function
ETEODL (Proposed)		97.38	0.977	0.977	0.96	0.95	28.63	

Table 2 shows the comparison of the proposed framework and conventional ML algorithms.

The outcome of the comparison in Table 2 can be concluded as follows:

- The DL-based framework outperforms traditional algorithms for the early detection of diabetes (21).
- The Extra-Tree ensemble feature extraction technique prevents the overfitting of the DL model (28).

**Figure 2:**
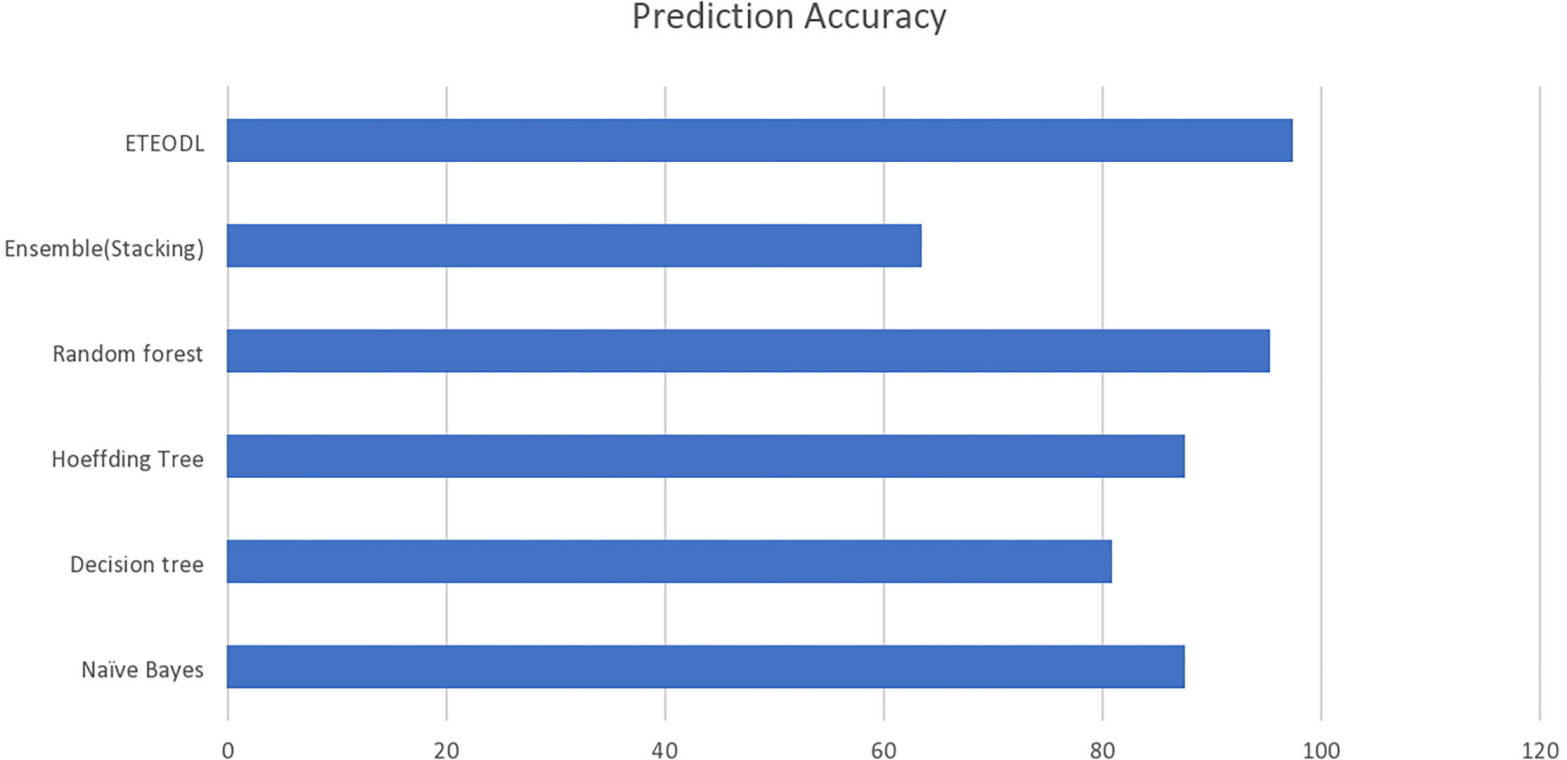
Accuracy (%) comparison with existing methods.

The graph in Figure 2 compares the prediction accuracy of conventional ML algorithms with the ETEODL (proposed).

**Figure 3:**
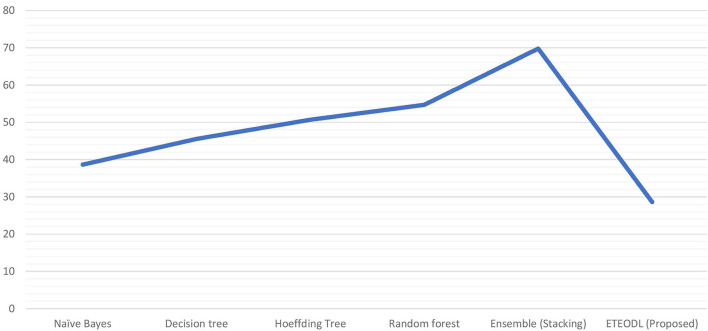
Comparison of computation time (in sec).

Figure 3 represents the graphs comparing the computation time of conventional ML algorithms with the ETEODL (proposed).

**Figure 4:**
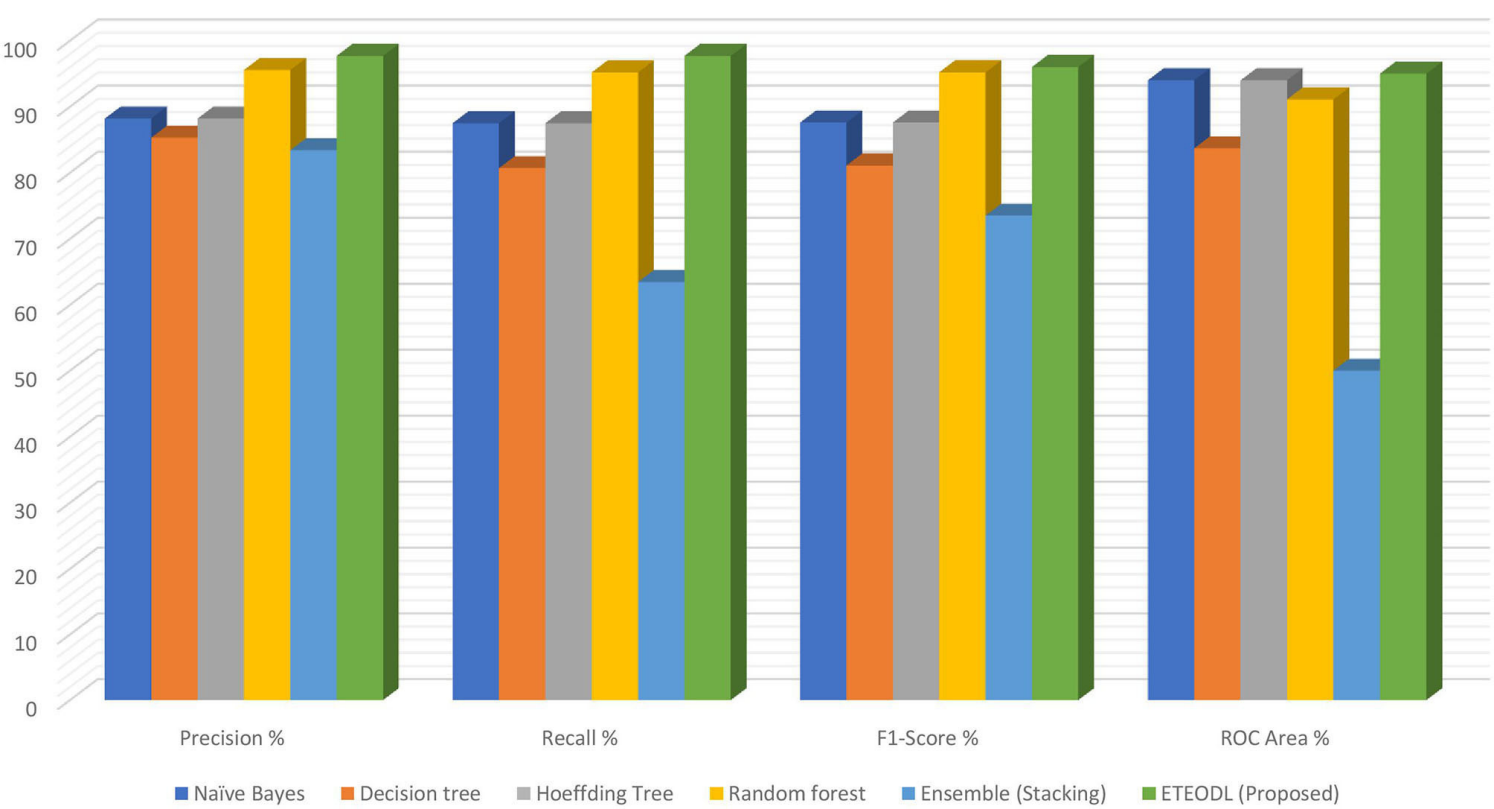
Comparison of precision, recall, F1-score, and ROC area.

Figure 4 represents the graphs comparing the precision, recall, and F1 score.

Table 3 shows the performance comparison of the proposed framework with Recent work.

The proposed work is less computationally complex and requires less computation time than previous related work while improving performance.

**Figure 5:**
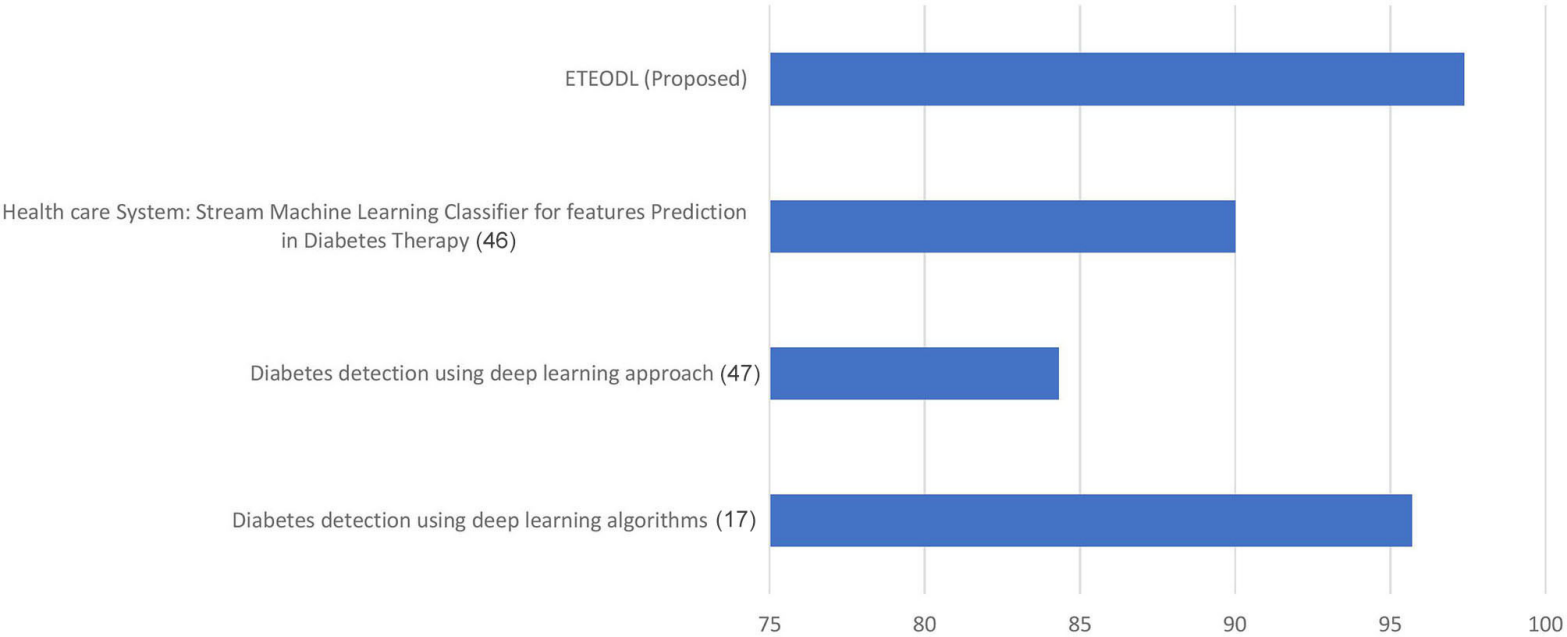
Comparison of prediction accuracy.

Figure 5 represents the graph comparing the accuracy of the proposed model with Recent works (17, 46, 47).

**Figure 6:**
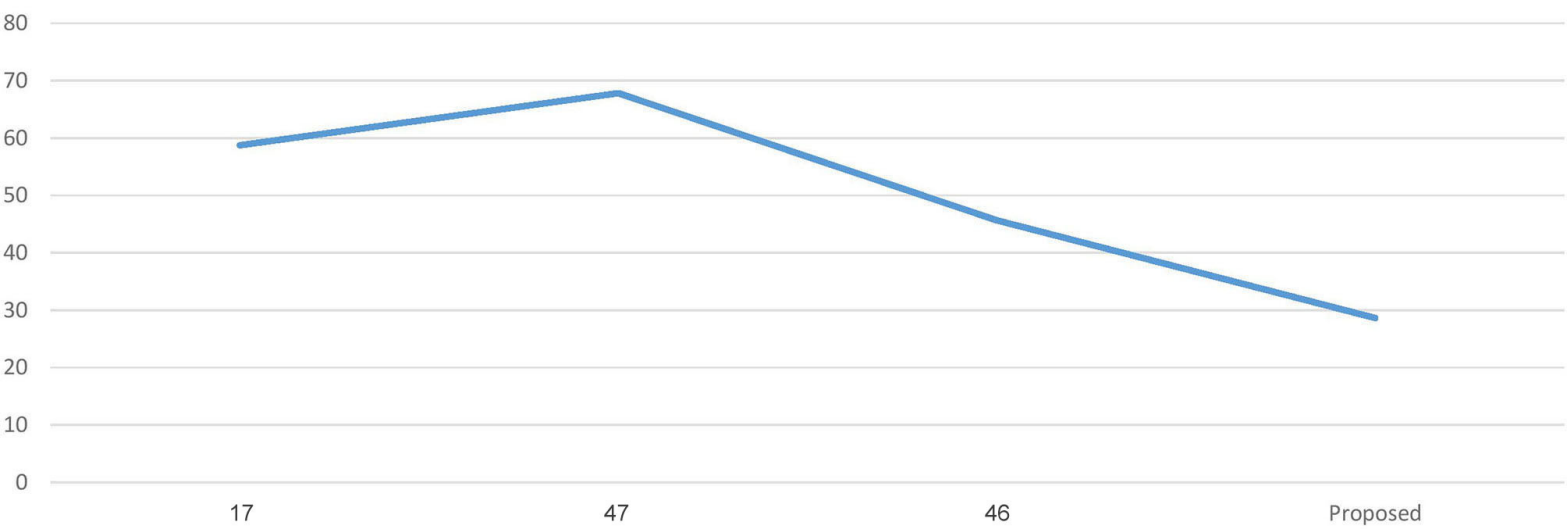
Comparison of computation time.

Figure 6 represents the graph Comparing the computation time of the Proposed model with three Recent works.

**Figure 7:**
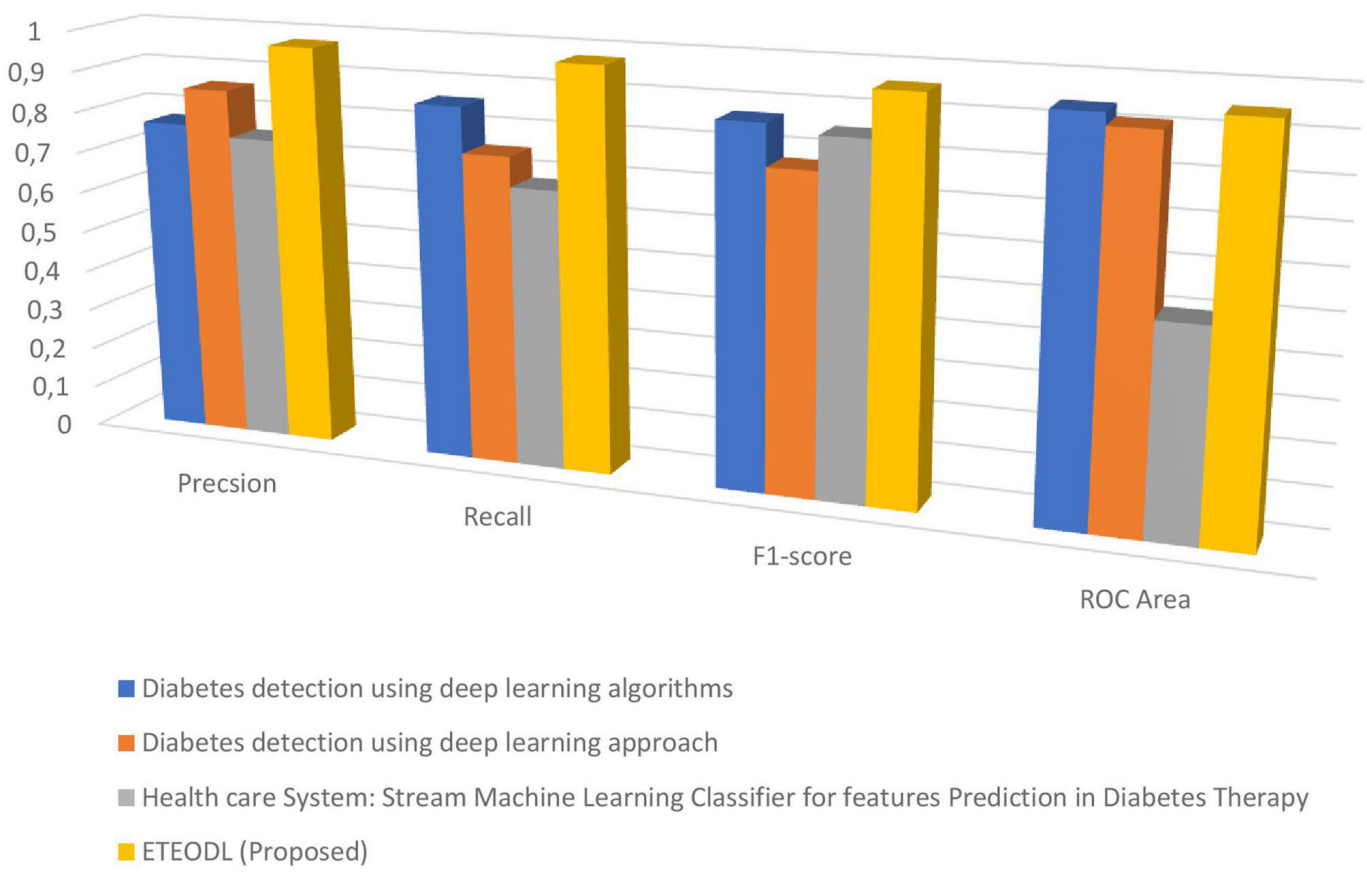
Comparison of precision, recall, f1-score, and roc area.

Figure 7 represents the graph Comparing the proposed model's Precision, Recall, and F1 score with Recent works.

Table 4 compares the training and testing accuracy of ETEODL over hundred epochs.

**Table 4:** Comparison of training and testing accuracy of ETEODL over 100 epochs.

**EPOCHS**	**Average training accuracy**	**Average testing accuracy**
01–10	0.836	0.954
11–20	0.877	0.962
21–30	0.922	0.968
31–40	0.934	0.971
41–50	0.952	0.973
51–60	0.964	0.976
61–70	0.965	0.977
71–80	0.966	0.977
81–90	0.968	0.978
91–100	0.968	0.978

**Figure 8:**
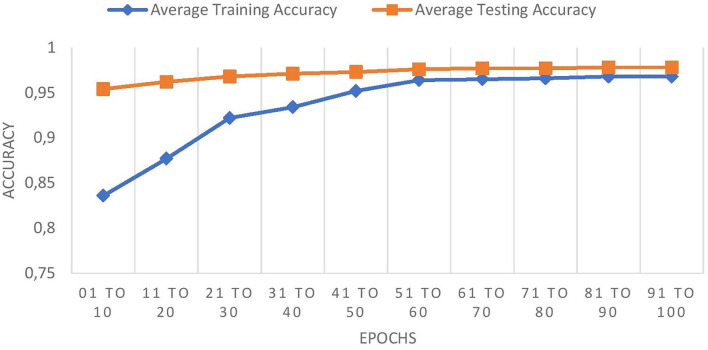
Comparison of training and testing accuracy.

The graph in Figure 8 shows the comparison of training and testing accuracies.

The comparison graph shows that the proposed work prevents overfitting as it maintains good training and testing accuracies.

## Conclusion

Detecting diabetes at an early stage is very consequential to take well-timed steps to avoid other health risks and complications. The proposed work demonstrates the application of the Extra Tree ensemble for optimization and DL for classification and prediction for diabetes. The proposed ETEODL is compared against the conventional ML techniques and ensemble algorithms and similar recent works. The accuracy obtained using ETEDOL was approximately 97.38% which is better than contemporary and traditional techniques. The information thus predicted can provide a caution signal for the patient and the doctor to take precautions and control measures. The proposed method is limited to numerical and categorical data and can also be developed for image data. In the future, the proposed model can be extended as an integral part of an automated system for diabetes prediction.

## Data Availability Statement

The original contributions presented in the study are included in the article/supplementary materials, further inquiries can be directed to the corresponding author.

## Author Contributions

MA and HS developed and implemented algorithm. AM, SK, and MA evaluated the performance of the algorithm and created the first draft. AZ further evaluated and benchmarked the work. HS and AZ finalized the manuscript. All authors contributed to the article and approved the submitted version.

## Funding

This work was supported by Taif University Researchers Supporting Project Number (TURSP-2020/114), Taif University, Taif, Saudi Arabia.

## Conflict of Interest

The authors declare that the research was conducted in the absence of any commercial or financial relationships that could be construed as a potential conflict of interest. The reviewer PK has declared a shared parent affiliation with the author AM at the time of review.

## Publisher's Note

All claims expressed in this article are solely those of the authors and do not necessarily represent those of their affiliated organizations, or those of the publisher, the editors and the reviewers. Any product that may be evaluated in this article, or claim that may be made by its manufacturer, is not guaranteed or endorsed by the publisher.
